# *Aphidius ervi* Teratocytes Release Enolase and Fatty Acid Binding Protein Through Exosomal Vesicles

**DOI:** 10.3389/fphys.2019.00715

**Published:** 2019-06-19

**Authors:** Rosanna Salvia, Annalisa Grimaldi, Rossana Girardello, Carmen Scieuzo, Andrea Scala, Sabino A. Bufo, Heiko Vogel, Patrizia Falabella

**Affiliations:** ^1^Department of Sciences, University of Basilicata, Potenza, Italy; ^2^Department of Biotechnology and Life Sciences, University of Insubria, Varese, Italy; ^3^Department of Geography, Environmental Management & Energy Studies, University of Johannesburg, Johannesburg, South Africa; ^4^Department of Entomology, Max Planck Institute for Chemical Ecology, Jena, Germany

**Keywords:** teratocytes, *Aphidius ervi*, enolase, *Aphidius ervi*, fatty acid binding protein, vesicles, exosomes, unconventional protein secretion

## Abstract

The molecular bases of the host-parasitoid interactions in the biological system *Acyrthosiphon pisum* (Harris) (Homoptera, Aphididae) and *Aphidius ervi* (Haliday) (Hymenoptera, Braconidae) have been elucidated allowing the identification of a gamma-glutamyl transpeptidase, the active component of maternal venom secretion, and teratocytes, the embryonic parasitic factors responsible for host physiology regulation after parasitization. Teratocytes, cells deriving from the dissociation of the serosa, the parasitoid embryonic membrane, are responsible for extra-oral digestion of host tissues in order to provide a suitable nutritional environment for the development of parasitoid larvae. Teratocytes rapidly grow in size without undergoing any cell division, synthesize, and release in the host hemolymph two proteins: a fatty acid binding protein (*Ae-*FABP) and an enolase (*Ae*-ENO). *Ae-*FABP is involved in transport of fatty acids deriving from host tissues to the parasitoid larva. *Ae*-ENO is an extracellular glycolytic enzyme that functions as a plasminogen like receptor inducing its activation to plasmin. Both *Ae*-FABP and *Ae*-ENO lack their signal peptides, and they are released in the extracellular environment through an unknown secretion pathway. Here, we investigated the unconventional mechanism by which teratocytes release *Ae*-FABP and Ae-ENO in the extracellular space. Our results, obtained using immunogold staining coupled with TEM and western blot analyses, show that these two proteins are localized in vesicles released by teratocytes. The specific dimension of these vesicles and the immunodetection of ALIX and HSP70, two exosome markers, strongly support the hypothesis that these vesicles are exosomes.

## Introduction

Teratocytes are specialized cells derived from the embryonic serosal membrane dissociation during parasitoid egg hatching in some hymenopteran endoparasitoid species (Dahlman and Vinson, 1993; Hotta et al., 2001; Strand, 2014). Teratocytes, together with other parasitic factors of maternal origin, such as polydnaviruses, venom, and ovarian proteins, are the “arsenal” of the parasitism success (Burke and Strand, 2014). Teratocytes rapidly grow in size without cell division, become highly polyploid, and release molecules impacting physiology, development, and nutritional suitability of colonized hosts (Dahlman and Vinson, 1993; Dahlman et al., 2003; Beckage and Gelman, 2004; Pennacchio and Strand, 2006). These large cells are characterized by a microvillar cell membrane, abundant endoplasmic reticulum, and numerous mitochondria, indicating that they are metabolically active (Pennacchio et al., 1994; Zhang et al., 1994). Teratocytes have been shown to synthesize and secrete a variety of proteins at all stages of parasitoid development (De Buron and Beckage, 1997). The nutritional role of teratocytes is generally mediated by digestion of selected host tissues, which release nutrients in a suitable form for the developing larvae (Falabella et al., 2000, 2009; Nakamatsu et al., 2002; Strand, 2014).

In the host-parasitoid system *Acyrthosiphon pisum* (Harris) (Homoptera, Aphididae) and *Aphidius ervi* (Haliday) (Hymenoptera, Braconidae), the cells of the serosal membrane dissociate into free teratocytes as soon as the parasitoid larva emerges in the host hemocoel. Five days after oviposition, 30 ± 5 free teratocytes can be observed in the host. After release, their number remains almost the same up to 7 days after parasitization and then decreases drastically to 18 ± 5 and 2 ± 1 teratocytes on days 8 and 9, respectively (Sabri et al., 2011). When released, the teratocytes are 40 ± 10 μm in diameter and increase in size during the two following days up to approximately 200 μm. At the same time, the cytoplasm fraction becomes progressively crowded with vesicles (Sabri et al., 2011).

Several proteins are synthesized and secreted by *A. ervi* teratocytes into the host hemocoel, and among them, two proteins with an apparent molecular mass of about 15 and 45 kDa are found in high abundance in the hemolymph of parasitized hosts (Falabella et al., 2000). These proteins lack the signal peptide, and they have been characterized as a fatty acid binding protein (*Ae*-FABP) (Falabella et al., 2005) and an enolase (*Ae*-ENO), respectively (Falabella et al., 2009; Grossi et al., 2016).

*Ae*-FABP shows a high affinity for C14-C18 saturated fatty acids (FAs) and for oleic and arachidonic acids (Falabella et al., 2005). The immunolocalization profile of *Ae*-FABP showed that it is distributed around lipid particles, abundantly present in the hemocoel of parasitized host aphids and in the midgut lumen of parasitoid larvae, suggesting that the protein acts essentially as a vector in host hemolymph, transferring FAs from the sites of host lipid digestion to the growing parasitoid larvae (Caccia et al., 2012). Enolase plays an important role in the glycolytic pathway (Walsh et al., 1989). When present in the extracellular environment on the surface of prokaryotic and eukaryotic cells, enolase evidences a main role in the activation of enzymes that allow the invasion of tissues by pathogens and tumor cells (Liu and Shih 2007; Marcilla et al., 2012; Silva et al., 2014). *Ae*-ENO is an extracellular enolase that contributes to the success of parasitism, acting as a receptor able to bind a host plasminogen-like molecule, which, once activated, induces the digestion of the host tissues. *In vivo* immunodetection experiments have shown that *Ae*-ENO is localized in round spots in the teratocyte cytoplasm and released into the host hemocoel, resulting in strong immunoreactivity in host embryos (Falabella et al., 2009). Accumulation of *Ae*-ENO in cytoplasmic vesicles oriented toward the cell membrane is observed followed by protein release outside of the cell. These vesicles seem to be localized on the cellular plasma membrane especially in microvilli, probably allowing the release of *Ae-*ENO into the extracellular space (Grossi et al., 2016).

Here, we demonstrate a possible secretory mechanism for *Ae-*ENO, extensible also to *Ae*-FABP, mediated by vesicles acting as mean of exocytosis, which was already speculated by Grossi et al. (2016). This mechanism seems to be shared by some micro-organisms that use an extracellular enolase to invade host tissues (Chavez-Munguia et al., 2011) and by several parasitic helminths releasing proteins, including enolase, through extracellular vesicles (Marcilla et al., 2012). Furthermore, we demonstrate that these vesicles are exosomes, mediating the extracellular transport of proteins lacking a signal peptide. We propose a non-canonical protein transport mechanism that represents the model of a possible strategy shared by different cell types and organisms involved in such processes.

## Materials and Methods

### Insect Rearing

*Aphidius ervi* was reared on the host *Acyrthosiphon pisum* maintained on plants of *Vicia faba* L. Both insect cultures were started with material originally collected on alfalfa plants, in Southern Italy. Aphids and parasitoids were kept in separate environmental chambers both at 20 ± 1°C, relative humidity of 75 ± 5%, and an 18:6 light/dark photoperiod. Experimental third instar aphids were singly exposed in a glass vial to a few *A. ervi* females and returned to plants until dissection.

### Teratocyte Collection and Stimulation

Teratocytes were collected as described by Falabella et al. (2000) from pea aphids, 5 days after parasitization by *A. ervi* and cultured in 1× phosphate-buffered saline (PBS) (pH 7.2). Briefly, aphids were dissected, and the released teratocytes were transferred into a 1.5 ml tube (Eppendorf, Hamburg, Germany) containing ice-cold PBS and allowed to settle for approximately 5 min on ice. The medium was then removed and replaced with an equal volume of PBS and teratocytes re-suspended by gentle swirling of the tube. This washing procedure was repeated at least three times.

Since ATP, through activation of the P2P7 ATP receptor, massively increases the release of vesicles (Bianco et al., 2005, Drago et al., 2017), teratocytes were exposed to 5 mM of ATP in PBS for 30 min to induce the production and release of vesicles in the extracellular environment.

### Light Microscopy

Samples were collected 5 and 6 days after *A. ervi* female oviposition (as described above, paragraph 2.1). Briefly, each parasitized aphid was dissected in a Petri dish in 100 μl 1× PBS. A stereo microscope (Nikon, Tokyo, Japan) was used for dissections, and *A. ervi* larvae (5 days after parasitization) and teratocytes (5 and 6 days after parasitization) were carefully transferred in a drop of 1× PBS and then observed by a Nikon Eclipse 80i at 10× magnification. Images were recorded by a Nikon Digital Sight DS-U1 camera (Nikon, Tokyo, Japan).

### Vesicle Isolation and Collection

After ATP exposure, teratocytes were gently removed using a micropipette and fixed for subsequent analyses, as described below. The incubation medium was subjected to differential centrifugation at 4°C as follows: 300 *g* for 5 min in order to remove debris, the obtained supernatant was then centrifuged at 1,200 g for 20 min, in order to separate any microvesicles (in the pellet) from exosomes (in supernatant). The exosome-rich supernatant was further centrifuged at 120,000 *g* for 1 h at 4°C in an ultracentrifuge (Beckman Coulter, Brea, CA, USA). The pellet, supposedly containing exosomes, was either resuspended in Laemmli buffer (Laemmli, 1970) for western blotting, or in 200 μl of ice-cold 1× PBS for further immunogold staining.

### Scanning Electron Microscopy

To obtain 3D imaging by scanning electron microscopy (SEM), teratocytes were collected from the parasitized hosts as already described (Falabella et al., 2009) and were attached to a small glass slide (9 mm × 9 mm) coated with 0.1% poly-l-lysine (Hotta et al., 2001). Subsequently, cells were fixed with Karnovsky fixative (2% paraformaldehyde and 2.5% glutaraldehyde) in 0.1 M cacodylate buffer (pH 7.2) for 1 h at room temperature. To obtain fine three-dimensional SEM imaging, an improved osmium maceration technique was employed (Franzetti et al., 2012). Teratocytes were fixed with 1% glutaraldehyde in 0.1 M Na-cacodylate buffer (pH 7.4) for 1 h at room temperature. After washes in Na-cacodylate buffer, specimens were post-fixed in a solution of 1% osmium tetroxide, 1.25% potassium ferrocyanide for 2 h. After several washings, cells were dehydrated in an increasing series of ethanol (70, 90, and 100%), and the small glass mounted on stubs gold and coated with a Sputter K250 coater (Emitech, Corato, Italy).

To obtain fine resolution of intracellular structures, teratocytes were fixed and post-fixed as described above, dehydrated in an increasing series of ethanol, embedded in an Epon-Araldite 812 mixture (Sigma-Aldrich, St. Louis, MO, USA), and sectioned with a Reichert Ultracut S ultramicrotome (Leica, Wetzlar, Germany). Semithin sections (700 nm in thickness) were collected on glass, and dried slices were treated with sodium methoxide to remove resin in excess. After mounting glasses on stubs, slices were subjected to critical point drying with CO_2_, gold coated with a Sputter K250 coater, and then observed with a SEM-FEG XL-30 microscope. All the samples were observed with a SEM-FEG XL-30 microscope (Philips, Eindhoven, The Netherlands).

### Transmission Electron Microscopy

For ultrastructural studies at TEM, teratocytes were recovered from hosts and dissected directly into fixative (0.1 M Na-cacodylate buffer at pH 7.4 containing 2% glutaraldehyde). After 2 h, cells were washed in the same buffer and post-fixed for 1 h with 1% osmium tetroxide in cacodylate buffer, pH 7.4. After standard serial ethanol dehydration, specimens were embedded in an Epon-Araldite 812 mixture (Sigma-Aldrich, St. Louis, MO, USA). Ultrathin sections (70–80) were obtained with a Reichert Ultracut S ultramicrotome (Leica, Wetzlar, Germany), collected on 200 mesh copper grids, counterstained with uranyl acetate and lead citrate, and observed by using a Jeol JEM-1010 electron microscope (Jeol, Tokyo, Japan) equipped with an Olympus Morada digital camera (Olympus, Tokyo, Japan).

### Glycogen Staining at Transmission Electron Microscopy

Teratocytes were stained by following the Thiéry method (1967). Cells were prepared by fixation in 2% glutaraldehyde, dehydrate in ethanol, and embedded in Epon araldite. Ultrathin sections (80–90 nm in thickness), obtained as above described, were collected on 200 mesh gold grids, incubated in 1% periodic acid in water for 30 min. After several washing in water, sections were treated with 1% thiosemicarbozide in 10% acetic acid, at RT for 30 min, washed in graded acetic acid series to water, stained for 30 min in 1% silver proteinate (Sigma-Aldrich, St. Louis, MO, USA) in double distilled water, washed in double distilled water. In control experiment, thiosemicarbozide treatment was omitted, and sections were incubated only with silver proteinate. All sections were counterstained only with aqueous uranyl acetate and observed by Jeol JEM-1010 electron microscope (Jeol, Tokyo, Japan). Images were recorded with the Olympus Morada digital camera (Olympus, Tokyo, Japan).

### Immunogold Labeling

For immunogold cytochemistry, teratocytes were fixed for 2 h with 4% paraformaldehyde and 0.5% glutaraldehyde in PBS, and then washed in the same buffer. After a standard step of serial ethanol dehydration, cells were embedded in an Epon-Araldite 812 mixture (Sigma-Aldrich, St. Louis, MO, USA) and sectioned with a Reichert Ultracut S ultramicrotome (Leica, Wetzlar, Germany). Ultrathin sections (80–90 nm in thickness) were collected on 200 mesh gold grids. After etching with 3% NaOH in absolute ethanol (Causton, 1984), sections were incubated for 30 min with PBS containing 2% bovine serum albumin (BSA) and then for 1 h at room temperature (RT), with the following primary antibodies (working dilution 1:40): rabbit polyclonal antibodies anti-Ae-ENO (Grossi et al., 2016) and anti-Ae-FABP (Falabella et al., 2005) and mouse monoclonal antibodies anti-Alix (Salvia et al., 2017) and anti-HSP70 (Sigma-Aldrich, St Louis, MO, USA). After several washing in PBS, samples were incubated for 1 h at RT with secondary goat anti-rabbit IgG (H + L)-gold conjugate (working dilution 1:100, particle size 6 nm, GE Healthcare Amersham, Buckinghamshire, UK) to detect Ae-ENO and anti-Ae-FABP, and with goat anti-mouse IgG (H + L)-gold conjugate (working dilution 1:100, particle size 6 nm, GE Healthcare Amersham, Buckinghamshire, UK) to detect Alix and HSP70, or with a protein G gold-conjugated 1:100, particle size 6 nm, GE Healthcare Amersham, Buckinghamshire, UK, to detect the mouse primary antibodies. The PBS used for antibody dilutions contained 2% BSA. In control sections, primary antibodies were omitted, and sections were treated with BSA containing PBS and incubated only with the secondary antibody goat anti-rabbit or with Protein G gold conjugated.

Isolated exosomes (obtained as above described) were prepared for immunogold staining (Thery et al., 2006). Briefly, 5 μl of exosome suspension was fixed in 50 μl of 2% paraformaldehyde. Five microliters of the mix were transferred onto 300 Mesh Formvar–carbon-coated electron microscopy grids (Pacific Grid Tech^1^). After 20 min, grids were transferred on drops of 100 μl of PBS with the sample membrane side facing down. After three washing in PBS, grids were pre-incubated for 30 min in blocking solution, and then immunostaining was performed as described above for teratocytes. All samples were counterstained with uranyl acetate in water and observed with a Jeol 1010 EX electron microscope (Jeol, Tokyo, Japan), and data were recorded with a MORADA digital camera system (Olympus, Tokyo, Japan).

### Western Blot

The exosome pellet obtained as described above was resuspended in Laemmli buffer (Laemmli, 1970) and loaded on SDS PAGE (12.5%). The two *A. ervi* proteins (Ae-ENO and Ae-FABP) were detected using antibodies anti-*Ae-ENO* (Grossi et al., 2016) and anti-*Ae*-FABP (Falabella et al., 2005) as primary antibody (both diluted 1:5,000 in 5% milk), and anti-rabbit conjugated to horseradish peroxidase (Life Technologies, Carlsbad, CA, USA) (diluted 1:5,000 in tris-buffered saline and 0.1% Tween 20 (TBS-T)), as secondary antibody. The two exosomes markers, Alix and HSP70 (Meckes and Raab-Traub, 2011), were detected by using Anti-Alix antibody, kindly provided by Dr. Toshiro Aigaki, and anti-HASP70 antibody (Sigma-Aldrich, St Louis, MO, USA) (diluted 1:5,000 in 5% milk). The anti-mouse conjugated to horseradish peroxidase as secondary antibody (Life Technologies, Carlsbad, CA, USA) was used diluted 1:15,000 in TBS-T. Finally, the Western blot Chemiluminescent HRP Substrate (ECL) (Luminata™ Crescendo, Western HRP Substrate, Millipore, Temecula, CA, USA) was used to detect target proteins, and signals were measured with Chemidoc™ MP System (Bio-Rad, Milan, Italy).

## Results

### Light Microscopy

**Figure 1:**
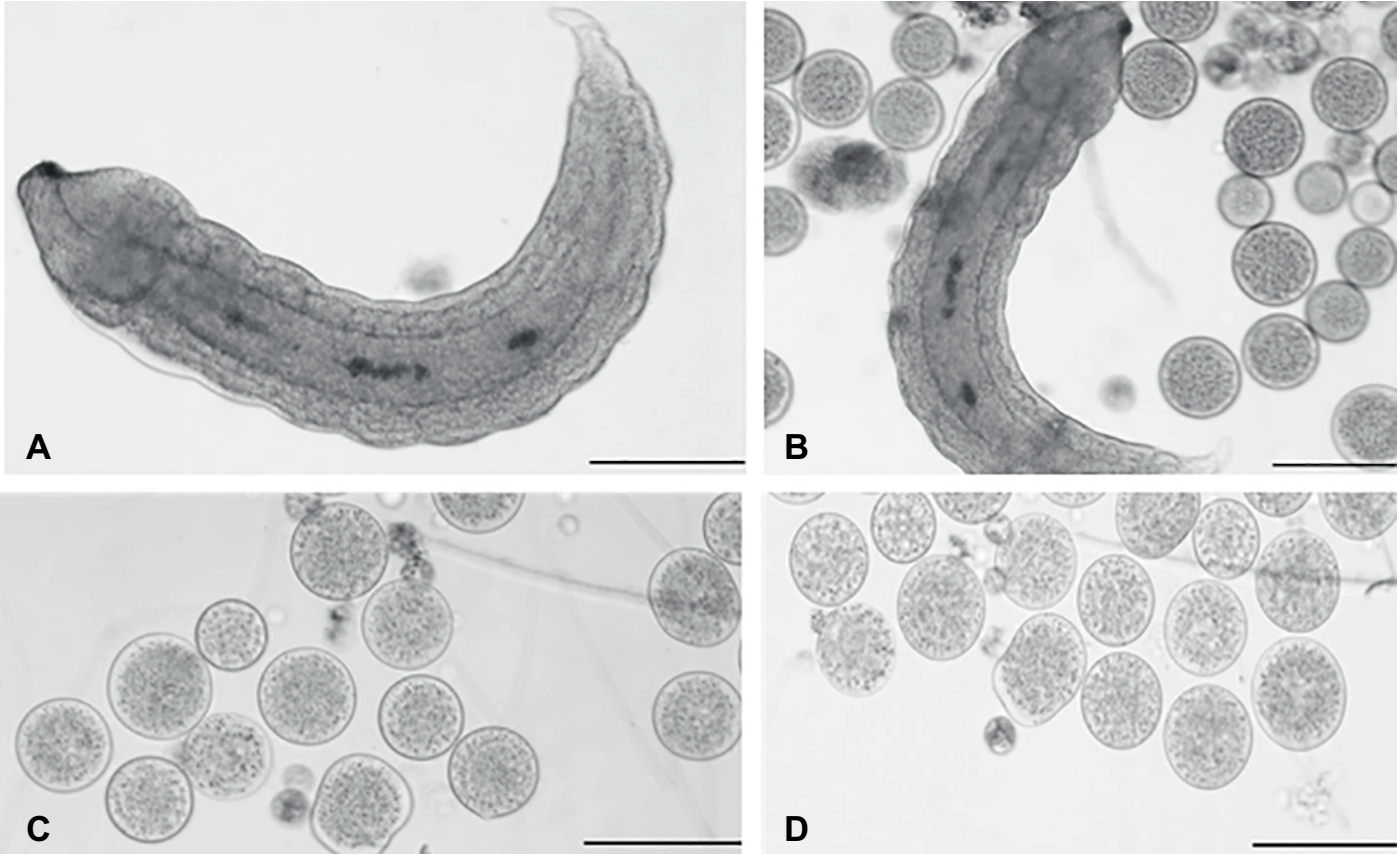
Light microscopy of teratocytes. *Aphidius ervi* larva **(A)**. Teratocytes and *Aphidius ervi* larva observed in the host hemocoel 5 days after parasitization **(B)**. Teratocytes observed at 5 days after parasitization **(C)**. Teratocytes observed at 6 days after parasitization **(D)**. A stereo microscope Nikon SMZ800 was used for dissections, and images were observed at a light microscopy using Nikon Eclipse 80i at 10× magnification. The images were recorded by Nikon Digital Sight DS-U1 camera and ImageJ software. Bars in **(A)**–**(D)**, 100 μm.

Teratocytes and *Aphidius ervi* larva were collected and observed after dissection of the host aphid, 5 and 6 days after parasitization (Figures 1A,B). After dissection, teratocytes were easily visualized, since they appeared as giant and buoyant spherical cells. As reported by Sabri et al. (2011), approximately 30 teratocytes in the hemocoel of each parasitized aphid dissected after 5 days following parasitization were counted. The same number was observed also 6 days after parasitization. Microscope observations showed that the size of teratocytes after their release was about 50 μm diameter (Figure 1C) and increased the following day to approximately 100 μm (Figure 1D).

### Morphological Analysis With Scanning Electron Microscopy

**Figure 2:**
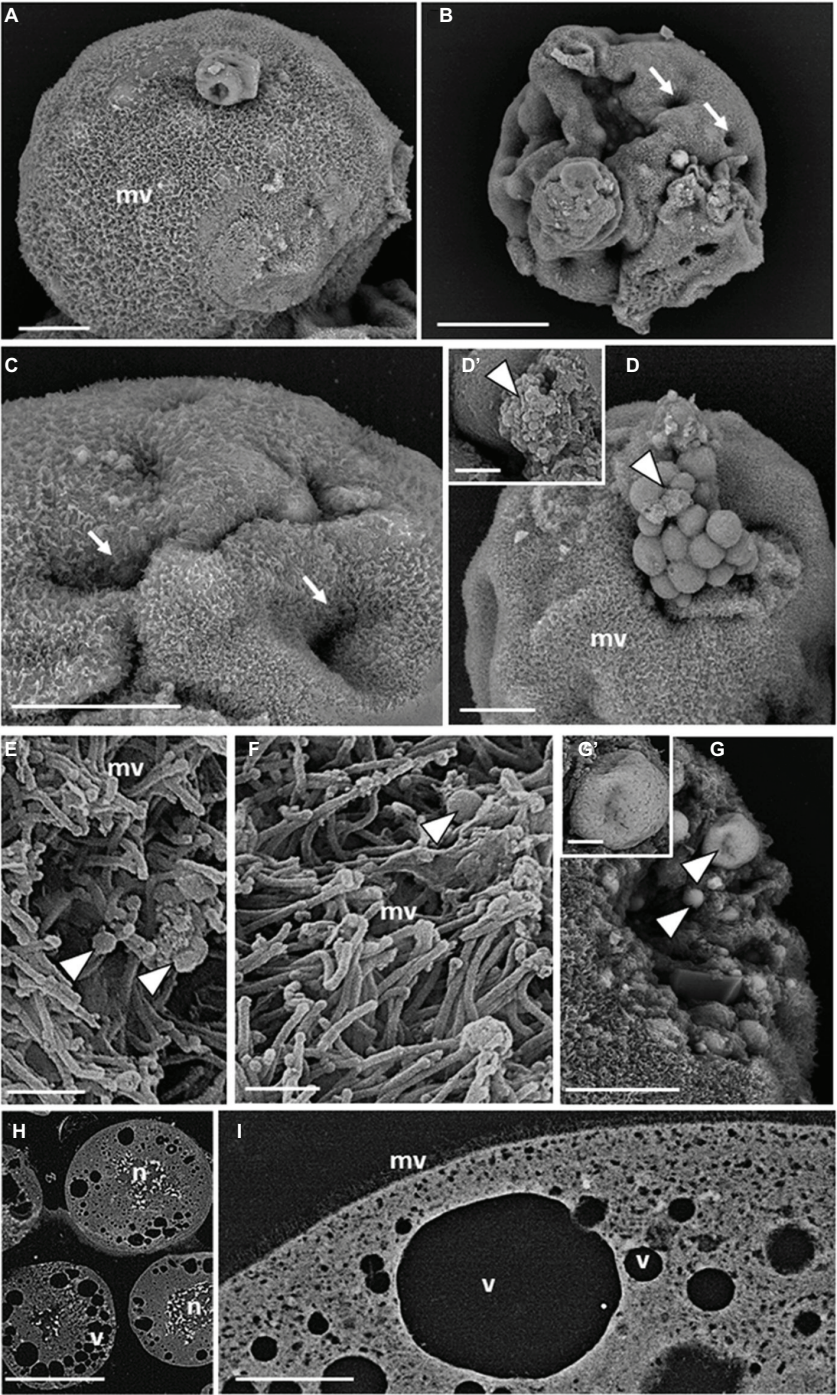
SEM micrographs of teratocytes. Teratocytes isolated from pea aphids 5 days after parasitization by *A. ervi* have a surface covered with numerous microvilli **(A)** and are characterized by the presence of indentations or dimple-like structures **(B,C)**. Spherical vesicles and structures similar to exosomes protrude from the plasma membrane [arrowheads in **(D,D′)**]. Numerous exosome-like structures [arrowheads in **(E,F)**] are interspersed among the microvilli. Spherical vesicles filled with smaller vesicles with circular profiles are visible in the cytoplasm [arrowheads in **(G,G′)**]. Large stellate nuclei **(H)** and vacuoles **(H,I)** are visible in the sectioned teratocytes. (mv) microvilli, (n) nucleus, (v) vacuoles. Bars in **(A,C,D,G)**: 20 μm; bar in **(B)**: 50 μm; bars in **(D′,G′)**: 2.5 μm; bars in **(E,F)**: 1 μm; bar in **(H)**: 100 μm; bar in **(I)**: 10 μm.

SEM revealed that the surface of teratocytes, collected 5 days after parasitization, was covered by a dense mat of microvilli (Figure 2A) and was characterized by the presence of indentations or dimple-like structures (Figures 2B,C). In addition, a blebbing phenomenon often became the dominant surface feature of some cells and spherical vesicles appeared on the external side of the microvilli-covered plasma membrane (Figure 2D). Smaller vesicular-shaped bodies, with a size ranging from 30 to 100 nm corresponding with the range of exosome-like structures, were grouped next to the large spherical vesicles (Figure 2D′) or interspersed among the microvilli (Figures 2E,F). Spherical vesicular formations of different sizes, limited by a membrane and filled with smaller vesicles with circular profiles (ranging size 50–100 nm), were also visible in the cytoplasm and underneath the plasma membrane (Figures 2G,G′). Analysis of sectioned teratocytes highlighted the presence of stellate nuclei (Figure 2H), localized in the center of the cytoplasm, and extended their ramifications toward the plasma membrane. Vacuoles of different sizes, occupying a substantial volume of the cytoplasm and in close proximity to the plasma membrane, were clearly evident (Figures 2H,I).

### Transmission Electron Microscopy: Morphological Analysis, Histochemical, and Immunogold Staining

**Figure 3:**
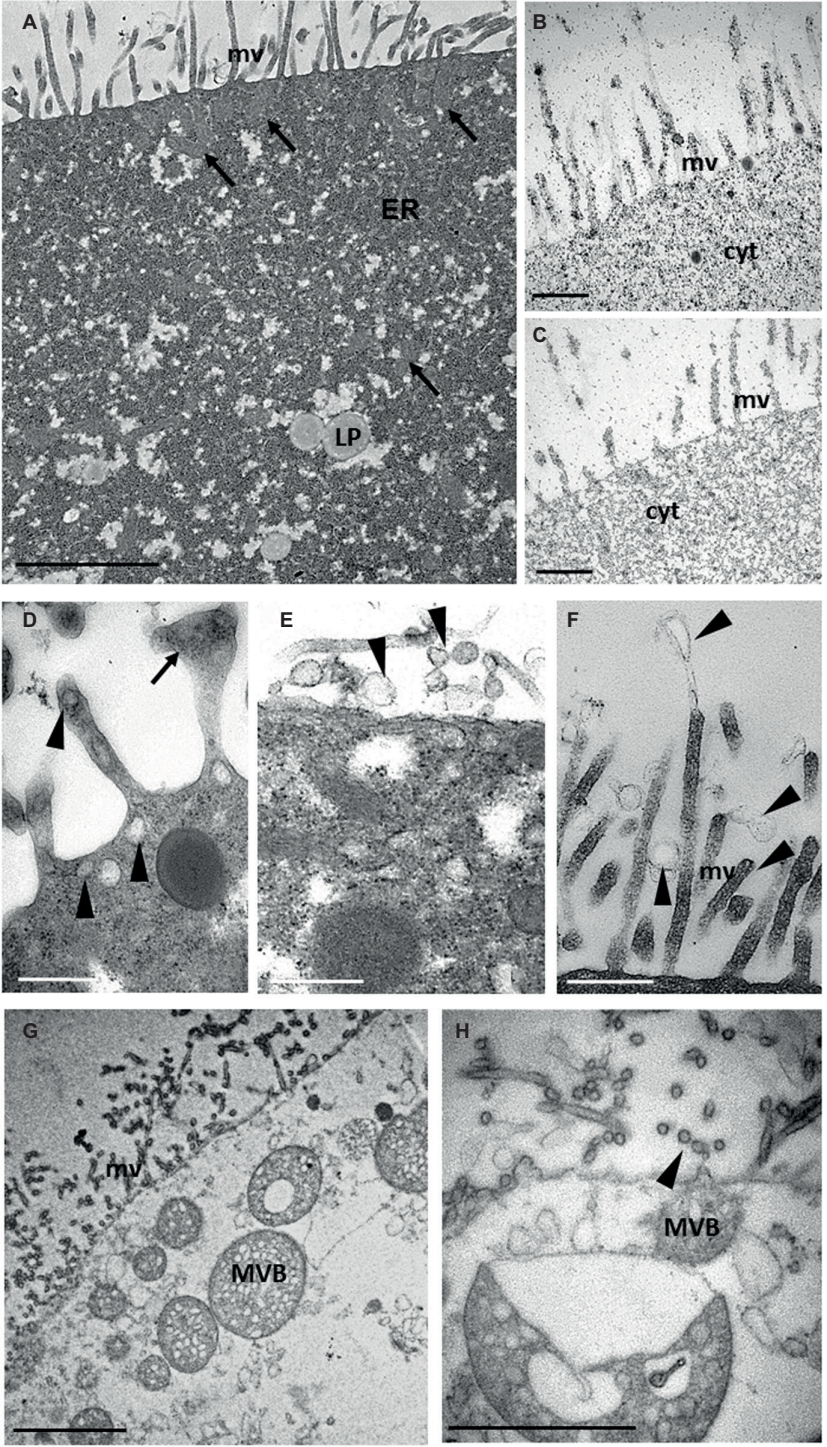
TEM ultrastructural analysis of teratocytes intracellular structures. A thick layer of microvilli (mv) covers the plasma membrane. Numerous mitochondria (arrows) are present under the coat of microvilli (arrows). Lipid droplets (LP) and abundant rough endoplasmic reticulum (ER) are visible in the cytoplasm **(A)**. Glycogen granules black stained by Thiery reaction are visible in the cytoplasm **(B)**. No black staining is detected in control sections **(C)**. Secretory vesicles (arrowheads) are visible in close proximity of the plasma membrane and of microvilli, some of which showing an enlarged tips (arrow) **(D)**. Secretory exosome-like structures (arrowheads) emanating from the plasma membrane **(E)** and from microvilli **(F)** can be observed. Multivesicular bodies (MVB) like structures having a single limiting outer membrane and containing internal vesicles of homogeneous size are visible in close proximity of plasma membrane **(G)**. Detail of a MVB exocyting exosome-like structures (arrowhead) **(H)**. Bars in **(A,G)**: 2 μm; bars in **(B–F)**: 500 nm; bar in **(H)**: 1 μm.

Further analyses of teratocyte intracellular structures were performed by ultrastructural analyses of thin sections with TEM. The plasma membrane formed a dense lawn of microvilli and underneath it, and numerous mitochondria with a regular shape were present (Figure 3A). Substantial amounts of rough endoplasmic reticulum (ER) and lipid droplets (LP) could be observed in the finely granular cytoplasm (Figure 3A). Such densely packed granules were shown to be glycogen by histochemical analysis (Figure 3B; Thiéry, 1967). No positively stained structures were found in non-oxidized thiocarbohydrazide-silver proteinate treated sections (Figure 3C), thus confirming that the cytoplasm of teratocytes contained a huge amount of glycogen. Taken together, these results suggest a possible specific role of teratocytes as a nutritional resource for the growth of parasitoid larvae. Indeed, several studies showed that braconid-wasp larvae consume teratocytes while feeding in the host hemocoel (Vinson and Iwantsch, 1980). Moreover, the accumulation of a great amount of glycogen and lipid in their cytoplasm confirmed that these cells had a high metabolic activity. These observations correlate well with the consistent secretory activity of these cells (Falabella et al., 2000). In fact, besides the presence of numerous vesicles (30–100 nm in size) mainly localized underneath the plasma membrane (Figure 3D), microvilli with enlarged tips (Figure 3D) as well as secretory exosome-like vesicles were observed along the external cell surface (Figure 3E), in close proximity to the microvilli and emanating from them (Figure 3F). Spherical organelles with a diameter of 250–1,000 nm, characterized by a single outer membrane enclosing a variable number of small spherical or ellipsoidal vesicles within a matrix and with heterogeneous sizes between 30 and 100 nm, were scattered in the cytoplasm and located in close vicinity of the plasma membrane (Figure 3G). Some of these organelles were exosome-like vesicles in the extracellular environment (Figure 3H). These structures, based on morphological criteria at the ultrastructural level, resembled the multivesicular body (MVB) organelles already described by several authors (Haigler et al., 1979; Cooney et al., 2002; Russell et al., 2006; Piper and Katzmann, 2007) and observed in almost all cell types of multicellular organisms.

**Figure 4:**
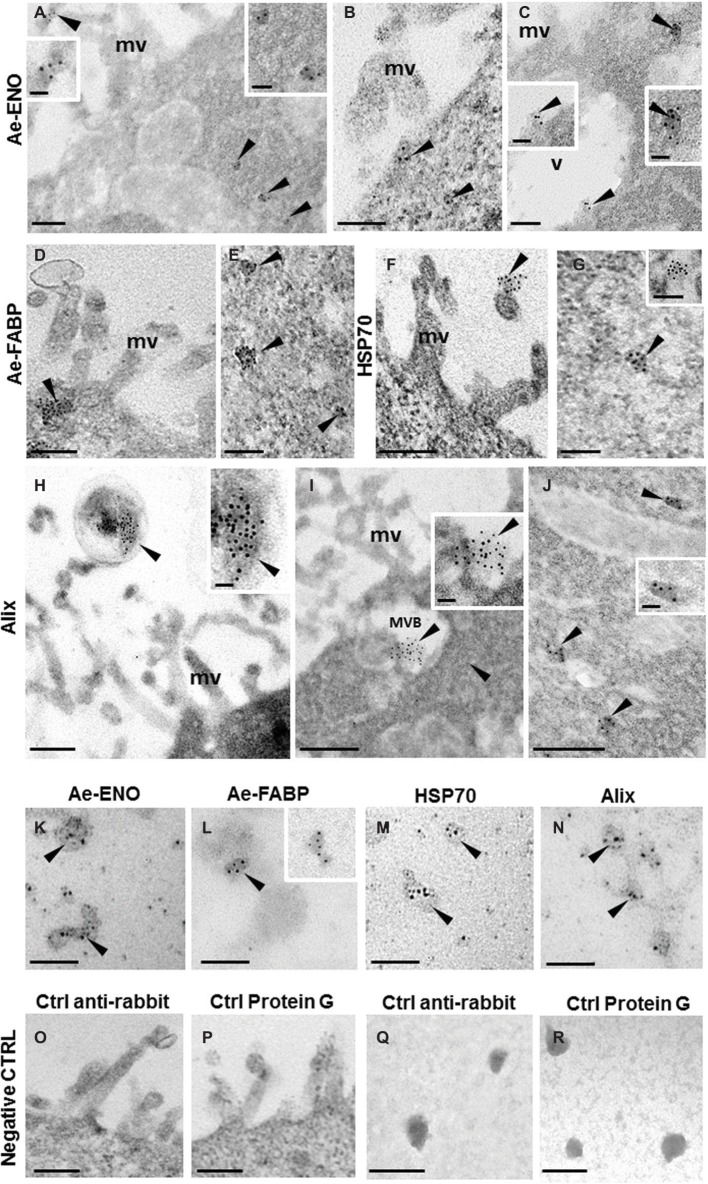
Immunogold staining at TEM of *Ae*-ENO, *Ae*-FABP HSP70, and Alix. Gold nanoparticles (arrowheads) are visible in exosome-like vesicles released from teratocyte microvilli **(A,F,H)** underneath the plasma membrane **(B–D)**, in the multivesicular body located next to the plasma membrane **(I)** and in those dispersed in the cytoplasm **(A,C,E,G,J)**. Immunostaining assays performed on exosome-like structures released by teratocytes stimulated for 30′ with ATP 5 mM **(K–N)**. No signal was detected in control experiments, where the primary antibodies were omitted, and samples were incubated only with the anti-rabbit secondary antibody **(O,Q)** or with the protein G gold-conjugated **(P,R)**. (mv) microvilli; (MVB) multivesicular body; (v) vacuole. Bars in **(A–G)** and in **(K–R)**: 100 nm; bars in **(H–J)**: 200 nm; bars in inserts 40 nm.

TEM immunogold labeling, performed on ultrathin sections of teratocytes, clearly showed that anti-*Ae*-ENO (Figures 4A–C and inserts), anti-Ae-FABP (Figures 4D,E), anti-HSP70 (Figures 4F,G and inserts), and anti-Alix (Figures 4H–J and inserts) antibodies marked exosome-like vesicles released from microvilli, located underneath the plasma membrane, in MVB or freely dispersed in the cytoplasm.

The same result was obtained by immunostaining assays performed on exosome-like structures released by teratocytes stimulated for 30′ with 5 mM ATP (Figures 4K–N). No signal was detected in control experiments (Figures 4O–R), the primary antibodies were omitted, and samples were incubated only with the anti-rabbit secondary antibody (Figures 4O,Q) or with the gold-conjugated protein G (Figures 4P,R).

### Western Blot

**Figure 5:**
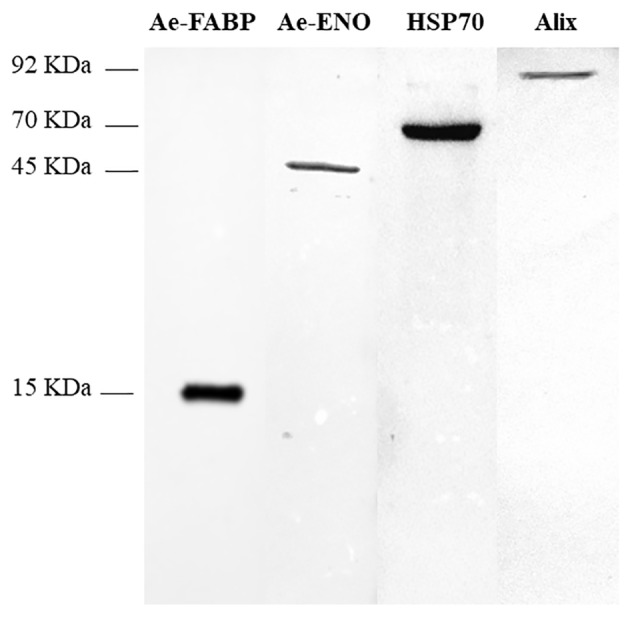
Western blot analyses performed on exosome-like vesicles released from teratocytes stimulated with ATP. The incubation with anti-*Ae*-FABP (15 kDa) and anti-*Ae*-ENO antibodies (45 kDa) confirmed that both proteins are released by vesicles. The incubation with anti-HSP70 (70 kDa) and anti-ALIX (92 kDa) antibodies, specific exosomal markers, strongly supports the hypothesis that vesicles are exosomes.

Western blot analyses were performed on exosome-like vesicles released by teratocytes stimulated with ATP, as described in the Materials and Methods section, paragraph 2.4. The signals detected with anti-*Ae*-FABP and anti-*Ae*-ENO antibodies (Figure 5, lanes 1, 2) unequivocally demonstrated that both proteins are localized in vesicles. Moreover, to confirm that collected vesicles are exosomes, anti-ALIX and anti-HSP70 antibodies, specific exosome markers, were used. Results strongly support the hypothesis that vesicles released by teratocytes are exosomes (Figure 5, lanes 3, 4).

## Discussion

Proteins destined to be secreted from cells or expressed at the cell surface usually enter the endomembrane pathway, mediated by the presence of a signal peptide that labels them for entering endoplasmic reticulum (ER) (Arnoys and Wang, 2007).

A number of secreted proteins without known secretion signals have been found (Kinseth et al., 2007), and several unconventional secretory pathways have been discovered or suggested (Nickel and Rabouille, 2009; Duran et al., 2010; Manjithaya et al., 2010). These proteins without typical secretion signals were shown to be involved in wound healing, cancer angiogenesis, cancer metastasis, inflammation, cytoprotection, and neurodegeneration (Lee et al., 2012; Pegtel et al., 2014; Bebelman et al., 2018; Groot Kormelink et al., 2018; Jabalee et al., 2018; Kawahara and Hanayama, 2018; Pinheiro et al., 2018; Rackov et al., 2018). In addition, these proteins are often released during stress conditions, and some of them have distinct intracellular and extracellular functions (Iraci et al., 2016). Therefore, it has been speculated that unconventional secretion might have developed early in the evolution before conventional secretion to rapidly connect diverse processes without going through extraordinary regulations (Radisky et al., 2009).

In this work, we investigated the unconventional mechanism of release of *Ae*-ENO and *Ae*-FABP proteins in the extracellular environment the hemocoel of parasitoid host. These two cytoplasmic proteins are synthetized and then released by *A. ervi* teratocytes, and their abundance suggests their critical role in the success of parasitism (Falabella et al., 2005, 2009). Fatty acid binding proteins (FABPs) belong to a family of small proteins (14–15 kDa), usually located in the cytosol and characterized by their ability to bind hydrophobic ligands non-covalently and with high affinity. FABPs are commonly involved in lipid metabolism by uptake and transport of long-chain fatty acids (FAs), and they interact with other transport systems and enzymes and are involved in signal transduction, gene expression, growth, and differentiation (Zimmerman and Veerkamp, 2002; Corsico et al., 2004). *Ae*-FABP is involved in transport of fatty acids from digestion sites of host lipids, through the host hemolymph, to the parasitoid larvae, and shows a particularly high affinity for C14–C18 saturated fatty acids (FAs) and for oleic and arachidonic acids (Falabella et al., 2005; Caccia et al., 2012).

Enolase is the eighth enzyme of the glycolytic pathway (Walsh et al., 1989) but is also defined as “moonlight protein” (Jeffery, 1999) since along with the classical role in glycolysis it also has several other functions, depending on its cellular location and/or cellular types (Aaronson et al., 1995; Subramanian and Miller, 2000; Gao et al., 2015; Ji et al., 2016). Among these different roles, enolase is an important extracellular protein related to different physiological and pathological processes. As extracellular protein enolase works as plasminogen receptor inducing its activation in plasmin, it is thus involved in tissue invasion processes. This strategy is shared by several different cellular types, both of eukaryotic and prokaryotic origin, such as parasites, yeasts, pathogenic bacterial cells, tumor cells, and cells belonging to the immune system, involved in physiological and pathological tissue invasion processes (Pancholi, 2001; Liu and Shih, 2007; Vanegas et al., 2007; Wygrecka et al., 2009; De la Torre-Escudero et al., 2010; Nogueira et al., 2010; Floden et al., 2011; Sanderson-Smith et al., 2012; Toledo et al., 2012; Ceruti et al., 2013; Godier and Hunt, 2013; Hsiao et al., 2013). In all these cases, enolase lacks its canonical signal peptide (required for entry into the secretory pathway), and thus, it is transported to the external cell surface through a mechanism not fully understood to date (López-Villar et al., 2006; Avilán et al., 2011; Ghosh and Jacobs-Lorena, 2011).

The extracellular localization of *Ae*-FABP and *Ae*-ENO is unusual, and the mechanism of release to the extracellular environment by teratocytes for both proteins has not been elucidated yet.

To date, four types of unconventional protein secretion mechanisms have been described: (1) direct transfer across the plasma membrane; (2) ABC transporter-based secretion for acylated peptides and yeast mating peptides; (3) underneath certain regions of the plasma membrane followed by release into the extracellular medium as a result of membrane blebbing and incorporation into exosomes; and (4) Golgi bypass pathway for proteins that enter the ER but bypass the Golgi apparatus to reach the plasma membrane (Nickel, 2003; Nickel and Rabouille, 2009; Rabouille, 2017).

Here, we demonstrate that *Ae*-ENO and *Ae*-FABP are released into the extracellular space by exosomal exocytosis.

According to the minimal experimental requirements for extracellular vesicle analysis recommended by the international Society for Extracellular Vesicles (ISEV) (Lötvall et al., 2014), we performed SEM, TEM, and western blot analyses on teratocytes and vesicles released *in vitro.*

Light microscopy observations showed that 5 days after *A. ervi* parasitization, serosa cells dissociate into around 30 free teratocytes with a size ranging from approximately 50 μm (5 days after parasitization) to about 100 μm (6 days after parasitization), according to the observations reported by Sabri et al. (2011). Ultrastructural analysis results showed that teratocytes exhibited numerous long and diffuse microvilli on their cell surface, and from them evaginated vesicular-shaped bodies with a size ranging from 30 to 100 nm, similar to exosome-like structures (Mathivanan et al., 2010), confirming the secretory activity of these cells (Falabella et al., 2000). We observed that both proteins, *Ae*-ENO and *Ae*-FABP, localized below the plasma membrane, in multivesicular bodies (MVBs) or freely dispersed in the cytoplasm, and in exosome-like structures released by teratocytes to the extracellular environment. Moreover, we collected exosomes released by teratocytes *in vitro,* and we detected the presence of both proteins by western blot analyses. In addition to the size range, specific exosome molecular markers are used to identify the exosomes and differentiate those from other vesicular structures. Exosomes contain a distinct set of proteins such as Alix, TSG101, HSP70, and the tetraspanins CD63, CD81, and CD9. In particular, Alix (identified in 68% of exosome studies) and HSP70 (89%) are highly associated with exosomes (Mathivanan et al., 2010). For this reason, we chose these two latter proteins as exosomes markers and verified their presence through immunogold staining, TEM observations, and western blot by using specific antibodies. HSP70 and Alix proteins are found in the cytoplasm of teratocytes, in their membrane blebs, and in the vesicles released from them. The results here presented confirmed that *Ae*-ENO and *Ae*-FABP are contained in exosomal like vesicles and led us to conclude that the unconventional secretion pathway of *Ae*-ENO and *Ae*-FABP could be effectively mediated by exosomes. Also, enolases, such as other enzymes associated with glycolysis (aldolase, GAPDH, etc.), are reported in ExoCarta^2^ as exosome markers. Also in helminths, among proteins secreted by exosome-like vesicles during infections, it has been identified an extracellular enolase (Bernal et al., 2006). Why exosomes contain so many glycolytic enzymes is unclear. It is possible that exosomes increase glycolytic activity in the target cells (Samoil et al., 2018). The unconventional secretion mechanisms of *Ae*-ENO and *Ae*-FABP are the same observed in two trematodes, *Echinostoma caproni* and *Fasciola hepatica*, which utilize exosome-like vesicles in the establishment of the infection of their hosts (Marcilla et al., 2012). Extracellular vesicles represent an important means of cellular communication transferring proteins, lipids, and genetic information (Mathivanan et al., 2010; Meckes and Raab-Traub, 2011; Willms et al., 2016). In eukaryotic cells, there are several extracellular organelles that are released, or shed, into the microenvironment (Mathivanan et al., 2010; Meckes and Raab-Traub, 2011). Across all eukaryotic kingdoms of life, extracellular vesicles have been pointed out as a ubiquitous mechanism for transferring information between cells and organisms. Their roles in normal physiology are not the only their remarkable point of interest. Extracellular vesicles mediate targeted intercellular communication under physiological and pathophysiological conditions. Parasite-derived extracellular vesicles can communicate information and transfer genetic material to host cells or other parasites (Mathivanan et al., 2010). Host-derived extracellular vesicles can play a key role in host defense and are candidates for generating a vaccine against pathogenic infection (Coakley et al., 2015).

Teratocytes of other parasitoid wasps, such as Braconidae, Aphididae, and Miramidae, are reported to secrete several proteins responsible for immunosuppression and inhibition of metamorphosis of hosts (Ali et al., 2013; Strand, 2014). Some of secreted proteins were identified and characterized: the teratocytes of *Microplitis mediator* have been shown to secrete collagens (Quin et al., 2000); *Microplitis croceipes* teratocytes secrete at least 15 different proteins, including TSP14, a protein with a signal peptide, that is involved in the developmental arrest of the host (Hoy and Dahlman 2002); *Cotesia plutellae* teratocytes release serpins and GTPase-activating proteins, both with signal peptides, involved in the inhibition of host cellular immunity (Ali et al., 2013); a chitinase, with signal peptide, released by teratocytes of *Toxoneuron nigriceps* was reported to promote the host cuticle digestion, allowing the parasitoid larva to emerge from the host body (Consoli et al., 2007). To date, the characterized proteins released by teratocytes of other parasitoid wasps seem to be secreted through a conventional mechanism involving the presence of signal peptide. To the best of our knowledge, this is the first study showing the mechanism of unconventional secretion of regulatory proteins released by teratocytes and lacking the signal peptide.

Enolase, in particular, is a protein of great interest, since it has been reported to be correlated to the progression of cancer and could represent a new therapeutic target (Dowling et al., 2007; Cappello et al., 2009, 2011); it was shown that enolase localizes to the surface of tumor metastasis cells and is responsible for the degradation of the extracellular matrix (Chang et al., 2006; Hsiao et al., 2013) similar to *A. ervi* enolase (Grossi et al., 2016). There is little evidence regarding the secretory pathway of enolase: *Saccharomyces cerevisiae* is reported to have autophagosome-mediated membrane compartments for the unconventional secretion of proteins (Bruns et al., 2011), or by SNARE-driven unconventional secretion (Miura et al., 2012). However, the mechanism by which enolase is transported to the surface of tumor cells remains unclear.

This work contributes to increase our knowledge regarding the unconventional mechanism of extracellular transport of proteins lacking canonical signal peptides. More generally, our study could contribute to clarifying the complex role of exosomal vesicles, more often considered as good diagnostic and drug-delivery tools, taking a central role in research of these fields, to understand the role that these vesicles play in the intercellular communication and to open new interesting scenarios for the regulation of physiological and pathological events.

## Data Availability

All datasets generated for this study are included in the manuscript.

## Author Contributions

PF designed the experiments, wrote, and critically revised the manuscript. SB and HV contributed to the data interpretation and critically revised the manuscript. RS performed light microscopy observations, collected vesicles, and prepared samples for subsequent SEM and TEM analyses. RS, AS, and CS performed the western blot experiments. AG and RG performed microscopy observations at SEM and TEM and immunogold labeling. All authors read and approved the manuscript.

### Conflict of Interest Statement

The authors declare that the research was conducted in the absence of any commercial or financial relationships that could be construed as a potential conflict of interest.

## Footnotes

^1^www.grid-tech.com

^2^http://www.exocarta.org/
